# Groundwater contamination risks and land use changes in a typical Agreste/Caatinga transition zone in northeastern Brazil

**DOI:** 10.1007/s10661-025-14961-z

**Published:** 2026-02-06

**Authors:** Emanuel Santos de Oliveira, Lanusse Salim Rocha Tuma, Celso Augusto Guimarães Santos, Manoranjan Mishra, Richarde Marques da Silva

**Affiliations:** 1 Graduate Program in Geography, Federal University of Paraíba, João Pessoa, Paraíba, 58051-900 Brazil; 2 Department of Geography, State University of Paraíba, Guarabira, Paraíba, 58200-000 Brazil; 3 Department of Civil and Environmental Engineering, Federal University of Paraíba, João Pessoa, Paraíba, 58051-900 Brazil; 4 Department of Geography, Fakir Mohan University, Vyasa Vihar, Nuapadhi, Balasore, Odisha 756089 India; 5 Department of Geosciences, Federal University of Paraíba, João Pessoa, Paraíba, 58051-900 Brazil

**Keywords:** Contamination, Google Earth Engine, Pollutants, Semiarid region, GOD method, POSH method

## Abstract

Effective management of limited and fragile groundwater resources is essential to ensure a sustainable, safe, and high-quality water supply. However, intensive anthropogenic activities are placing increasing pressure on groundwater systems worldwide. Groundwater contamination and changes in land use and land cover in water-scarce cities represent serious environmental challenges, particularly in the Agreste/Caatinga transition zone of northeastern Brazil. This study therefore assesses groundwater contamination risks and land use changes in the city of Campina Grande, located in northeastern Brazil. The following procedures were employed: (a) identification of wells through data collection from public agencies; (b) evaluation of the natural vulnerability to groundwater contamination using the Groundwater Overall Depth (GOD) method; (c) spatiotemporal analysis of land use and land cover changes using the Google Earth Engine platform; (d) identification of point sources of contamination and assessment of potential contamination risk using the Pollutant Origin and its Surcharge Hydraulically (POSH) method; and (e) mapping of groundwater vulnerability and contamination risk. The study area was classified into three distinct levels of natural vulnerability—low, medium, and high. The results indicate an increase in groundwater extraction beginning in the 1990s, particularly between 1995 and 2000, strongly correlated with drought periods. The findings also reveal rapid urban expansion and an increase in the number of pollution sources. Approximately 33% of the study area exhibits high vulnerability on the aquifer’s natural vulnerability map, as determined using the GOD index. It is concluded that land use changes and the associated risks of groundwater contamination in Campina Grande represent serious threats to environmental quality and water availability for the city.

**Graphical Abstract:**

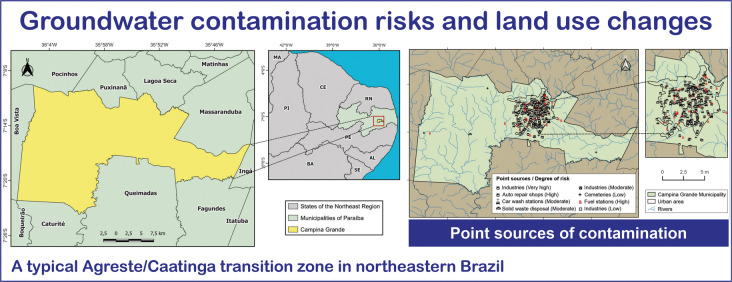

## Introduction

Groundwater plays a vital and strategic role in supporting socioeconomic development, particularly in semiarid regions where surface water resources are scarce or highly variable (Dlamini et al., 2025; Oliveira et al., 2022). In many such areas, groundwater often constitutes the only reliable and sustainable source of freshwater for human consumption, agriculture, livestock, and various industrial activities (Guria et al., 2024; Tôrres et al., 2025). Its importance is especially evident in cities historically vulnerable to water scarcity, where centralized water infrastructure is limited or nonexistent. This is the case of Campina Grande, the fifth largest city in Brazil’s semiarid region (Vidal et al., 2020). According to Silva et al. (2024), several drought periods have been recorded in Campina Grande at intervals of 5 to 10 years, resulting in recurring issues with water availability and severe supply crises—the most critical occurring in 2021, reportedly the most severe in the past 90 years (Silva et al., 2021).

Groundwater not only ensures water security for domestic needs but also sustains food production systems and economic resilience by enabling irrigation and industrial processes. Furthermore, its generally higher quality compared to surface water makes it a preferred option for drinking water supply and sanitation. Given the increasing pressures from climate change, population growth, and economic expansion, the sustainable management and protection of aquifer resources have become urgent priorities to ensure long-term water availability and equitable access for present and future generations (Adenova et al., 2025).

Both the quantity and quality of groundwater are directly influenced by land use practices (Ghoto et al., 2025). Population and economic growth, coupled with land use and land cover changes, are key drivers that affect groundwater quality (Kumar et al., 2025). As urban areas expand and agricultural and industrial activities intensify, pollutants such as sewage, fertilizers, and chemical waste increasingly infiltrate the soil layers, reaching aquifers (Souri et al., 2025). Improper land management and irregular waste disposal further exacerbate contamination risks.

Changes in land use and cover not only affect variables within the hydrological cycle but may also cause complications in urban drainage systems, increasing the frequency of floods. In addition, such changes lead to reduced evapotranspiration due to vegetation loss, decreased infiltration resulting from soil sealing, elevated contamination of surface and groundwater, and shifts in vegetation patterns in urban environments (Quddoos et al., 2024).

In Brazil, medium-sized cities have experienced a significant increase in population since the 1990 s and 2000 s, largely driven by rural-to-urban migration processes initiated in the 1970 s (Castiglioni, 2020). The growing reliance on groundwater extraction presents a concerning scenario regarding contamination risks. This process is often linked to the instability of surface water use. In this context, Brazil faces challenges related to the unequal distribution of water across intra- and inter-regional scales, involving both scarcity and abundance, as well as degradation due to domestic and industrial pollution. The most pronounced scenario of scarcity is observed in the semiarid region of northeastern Brazil (Hirata et al., 2025).

As in other countries, groundwater in Brazil represents a critical component of water security, particularly in the context of global climate change. Despite its essential role in urban supply, irrigation, and industry, groundwater management remains inadequate, and data on its use and on the impacts of insufficient sanitation infrastructure are scarce (Cambraia Neto et al., 2021). Monitoring and understanding land use changes and groundwater contamination vulnerabilities are essential for more informed and coherent water resource management. Over the past three to four decades, numerous methods have been developed to assess groundwater vulnerability to anthropogenic pollution (Machiwal et al., 2018; Ourarhi et al., 2022). These approaches have been widely adopted by researchers and policymakers to identify areas of particular vulnerability and to support the prevention of groundwater contamination (Ourarhi et al., 2024a). Among the most widely applied are the Groundwater Overall Depth (GOD) method, the Pollutant Origin and its Surcharge Hydraulically (POSH) method, and DRASTIC (Aller et al., 1987), which have been implemented in diverse regions (Fannakh & Farsang, 2022). Their popularity stems from their ease of application, reduced time requirements, and compatibility with GIS frameworks. These methods are based on the assumption that specific hydrological, geological, and climatological factors govern the potential for groundwater contamination (Ourarhi et al., 2024b).

This study employed four complementary techniques for mapping groundwater contamination risks and land use changes: (a) evaluation of natural vulnerability to groundwater contamination using the GOD method; (b) spatiotemporal analysis of land use and land cover changes using the Google Earth Engine platform; (c) identification of point sources of contamination and assessment of potential contamination risk using the POSH method; and (d) mapping of groundwater vulnerability and contamination risk. This integrative approach distinguishes the study as one of the first to analyze groundwater contamination risks and land use changes in a typical Agreste/Caatinga transition zone in northeastern Brazil. The integration of these techniques enables a comprehensive analysis of local conditions and provides a reproducible framework applicable to other regions. Such an approach enhances the capacity to manage groundwater pollution risks with greater precision and efficiency.

The selection of the GOD and POSH models to assess groundwater vulnerability and contamination risk was based on their technical characteristics and suitability to the local context. The GOD method was originally designed for regions with limited data availability and is widely used to evaluate intrinsic groundwater vulnerability by considering groundwater occurrence, overlying lithology, and depth to the water table (Ourarhi et al., 2024c). Its application is appropriate in areas with well-defined geological and hydrogeological datasets, such as the study area. Conversely, the POSH method is effective in assessing contamination risk by accounting for pollutant loading, soil characteristics, and regional hydrology (Campoverde-Muñoz et al., 2023). The use of POSH is particularly relevant in areas with intensive land use, such as the study region, where agricultural and urban activities can adversely affect groundwater quality (Taghavi et al., 2022). The combination of these methods enables a comprehensive analysis integrating intrinsic vulnerability and contamination risk, thereby supporting the sustainable management of water resources in the region. Therefore, this study aims to evaluate land use and land cover transformations and the associated risks of groundwater contamination in Campina Grande, a representative city situated in the Agreste/Caatinga transition zone of northeastern Brazil.

## Materials and methods

### Study area

**Fig. 1:**
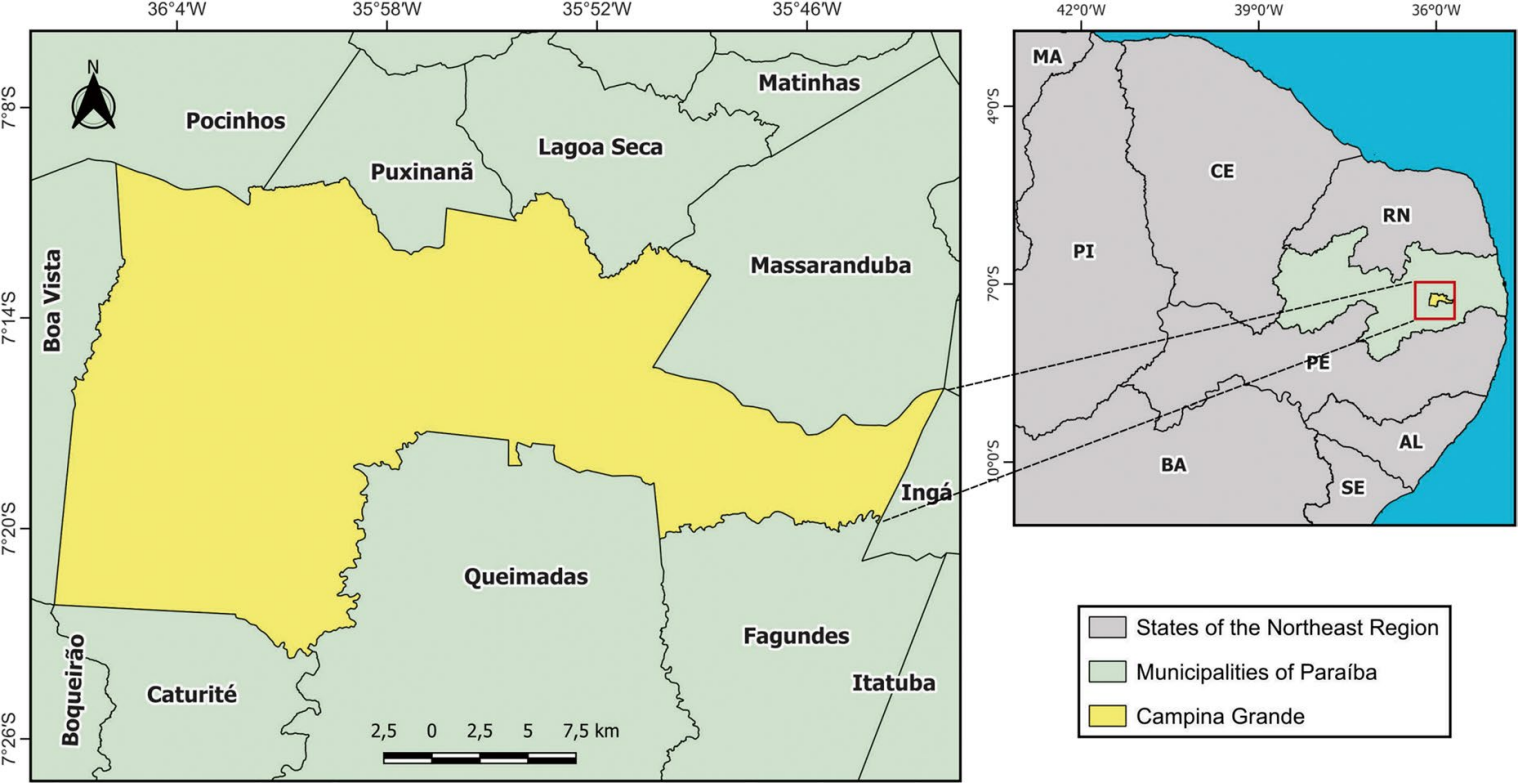
Geographical location of the municipality of Campina Grande

The study area comprises the municipality of Campina Grande, located between 7° 23′ 13″ S and 7° 9′ 15″ S latitude and 36° 7′ 25″ W and 35° 43′ 15″ W longitude (Fig. 1). Campina Grande covers a total area of 486.98 km^2^, with an urban area of approximately 66.64 km^2^. The municipality has a population of 419,379 inhabitants and a population density of 708.82 inhabitants per km^2^ (IBGE, 2022). Campina Grande plays a prominent role in the state of Paraíba due to its continuous industrial and commercial development, population growth, expansion of residential areas (both formal and informal), and increasing demand for basic sanitation services (de Araújo Neto et al., 2021).

#### Physiographic aspects

The topography of the study area lies within the eastern slopes and central pediplain of the Borborema Plateau. It is characterized by convex and tabular plateau forms, with altitudes ranging from 250 to 650 m (Dornellas et al., 2020). The municipality exhibits gently undulating relief, with the most rugged terrain located in the northeastern sector. In the southwestern region lies the Serra do Monte (a chain of inselbergs), while the southeastern portion features two alignments: the Serra de Catauma and Serra de Bodopitá (Lima et al., 2021).

The municipality comprises the following soil types: Eutrophic Regolithic Neosols, Eutrophic Litholic Neosols, Eutrophic Red-Yellow Argisols, Chromic Orthic Luvisols, and Orthic Natric Planosols (de Medeiros et al., 2019). The soils are generally shallow and exhibit clay-sandy textures, reflecting the region’s limited rainfall indices. As a result, the development of dense forests is constrained. Nevertheless, the vegetation is diverse, including the presence of cacti, legumes, and bromeliads, as well as sparse associations of native species such as *Ziziphus joazeiro* (juazeiro), *Prosopis juliflora* (algaroba), *Spondias tuberosa* (umbu tree), and *Sideroxylon obtusifolium* (marmeleiro) (Cordeiro et al., 2025).

According to the Köppen climate classification, the predominant climate type is AS’, characterized as hot and humid, with rainfall occurring mainly during autumn and winter, followed by a dry season lasting approximately 5 to 6 months (de Farias Panet et al., 2022). Precipitation is influenced by the Atlantic Equatorial air mass, which intensifies during the autumn, bringing higher humidity via the lower easterly trade winds. In winter, polar air masses from the south interact with the southeastern trade winds, producing significant rainfall, particularly along the coastal regions (Silva et al., 2020).

#### Geological and hydrogeological context

**Fig. 2:**
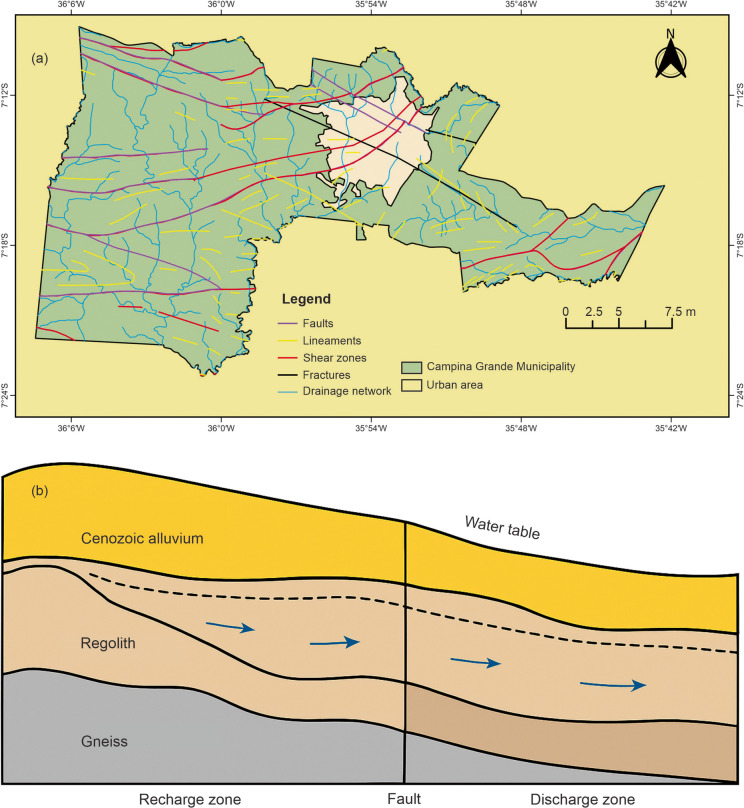
**a** Hydrogeological map of the Campina Grande municipality and **b** schematic cross-section showing the geological–hydrogeological setting of the study area

The study area is situated within two major geological domains: the Transversal Zone and the Rio Grande do Norte Domain. These domains are influenced by Brasiliano shear zones and Ediacaran granitic intrusions (Lima et al., 2024). The Transversal Zone encompasses nearly the entirety of the Precambrian lithotypes, whereas the Rio Grande do Norte Domain extends over a narrow strip in the northwestern portion of the mapped area, located north of the Patos Shear Zone. This zone is composed of Ediacaran-aged metasedimentary rocks belonging to the Seridó Group (Lima et al., 2021). The hydrogeological system in the study area is embedded within the crystalline basement, which is characterized as a discontinuous medium (Fig. 2). This condition results in randomly distributed aquifers, typically associated with saline groundwater. The salinization is primarily due to the lack of a weathered mantle, which, in turn, reflects the region’s low precipitation rates and high evaporation levels (Souza et al., 2025).

### Well mapping

**Fig. 3:**
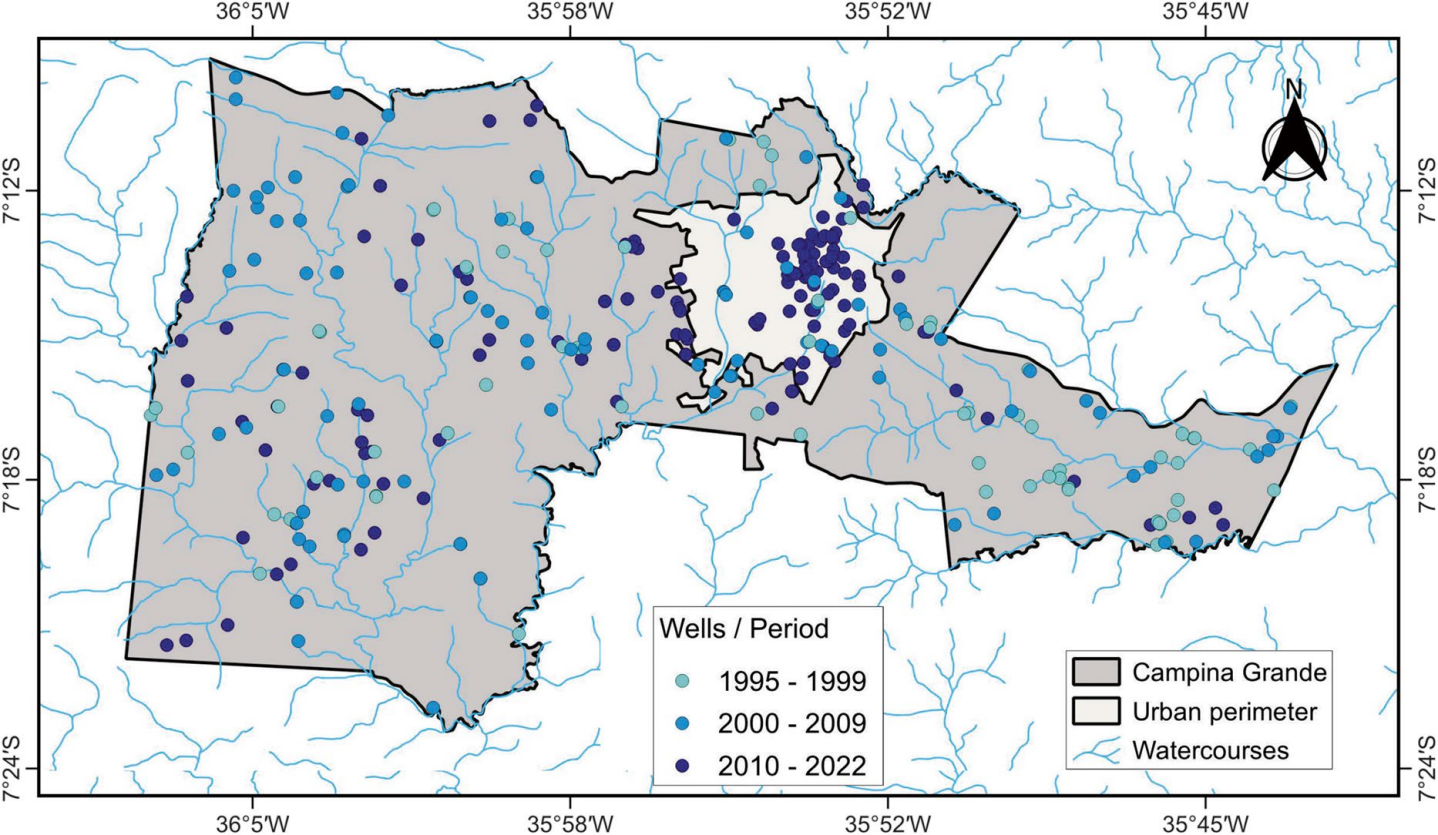
Spatial distribution of wells used in the groundwater contamination risk analysis

To analyze the potential sources of groundwater pollution, data on wells covering the period from 1995 to 2022 were used. These data were obtained from the licensing and permitting system of the Executive Water Management Agency of the State of Paraíba (AESA, 2024) and the online portal of the Groundwater Information System of the Geological Survey of Brazil (SGB, 2024). A total of 199 wells—both public and private—were mapped for various uses, including domestic supply, industrial and agro-industrial activities, commerce, recreation, and irrigation (Fig. 3). The selection and acquisition of well data were based on two criteria: (1) the time period during which the wells were drilled and (2) the issuance of permits or licenses for wells within the municipality. Temporal analysis was conducted by examining the historical drilling records. Subsequently, the data were spatialized using GIS techniques to assess the spatial distribution of wells within the study area. Each well included the following information: (a) determination of the groundwater confinement level, (b) lithological characteristics of the strata, and (c) depth to the water table.

### Contamination risk assessment

**Fig. 4:**
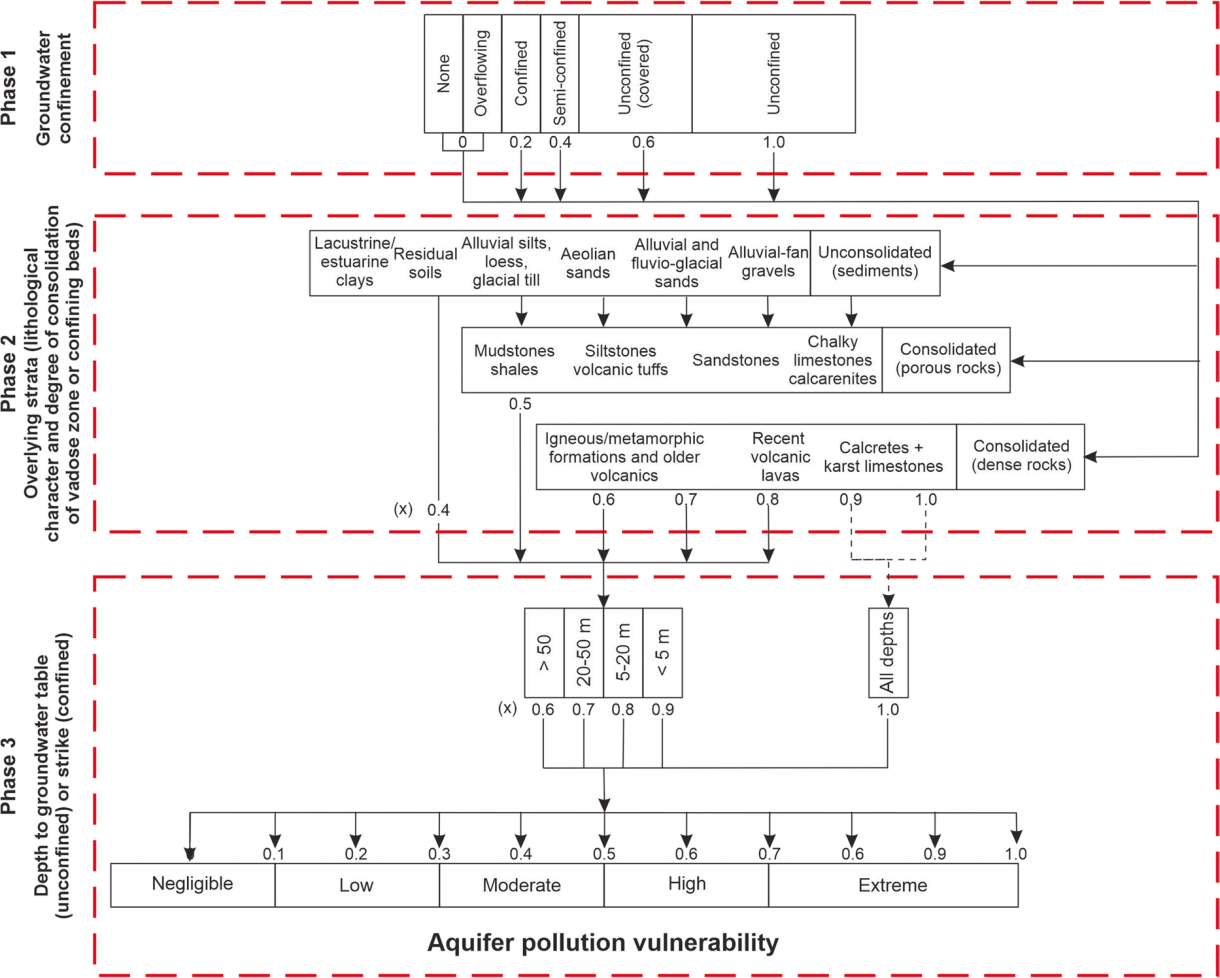
Schematic representation of the GOD method application

The methodology used to assess groundwater contamination risk was based on the application of the GOD method (Foster, 1987) (Fig. 4). This method comprises three main steps: determination of the degree of groundwater confinement (*G* parameter), with values ranging from 0.0 to 1.0, indicating the type and presence of aquifers in the region, including their storage capacity and flow characteristics; determination of the lithological characteristics of the unsaturated zone (*O* parameter), with values between 0.3 and 1.0, assessing the soil and rock types between the surface and the aquifer, which influence natural protection against contaminant penetration; and determination of the depth to the water table (*D* parameter), with values ranging from 0.6 to 1.0, considering the distance between the surface and the water table, where greater depths generally correspond to lower vulnerability. Each parameter is assigned a score or class based on predefined criteria, and the combination of these factors produces a vulnerability index, generally classified into categories of low, medium, or high vulnerability.

The GOD index is based on three main parameters and is calculated after assigning a value to each parameter, as follows (Eq. 1):

1 $$GOD = G \times O \times D$$

where *G* represents the type of groundwater occurrence (e.g., phreatic, semi-confined, or confined), *O* refers to the overall lithology of the unsaturated zone, and *D* corresponds to the depth to the water table. Table 1 presents the definitions associated with each groundwater vulnerability class according to the GOD method. Each parameter used in the GOD method was derived from well technical reports provided by the SIAGAS database, in accordance with the geological map of the Borborema Province and the hydrogeological map of the state of Paraíba. This classification encompasses a range of vulnerability scenarios and enables aquifer vulnerability to contamination to be categorized as insignificant, low, moderate, high, or very high.

**Table 1:** Practical definition of groundwater vulnerability classes

Vulnerability class	Corresponding definition	Value
Very high	Vulnerable to most contaminants with rapid impact under many contamination scenarios	8
High	Vulnerable to many contaminants (except those that are highly adsorbed or rapidly degraded) under many contamination scenarios	6
Moderate	Vulnerable to some contaminants, but only when continuously released or leached	4
Low	Vulnerable only to conservative contaminants over the long term when continuously and extensively leached	2
Very low	Presence of confining layers with no significant vertical groundwater flow (percolation)	1

### Identification of point sources of groundwater contamination

The Pollutant Origin and its Surcharge Hydraulically (POSH) method was applied to evaluate the potential occurrence of point sources of contamination that could affect groundwater quality (Foster et al., 2002). The POSH method is an index designed to assess the risk of aquifer contamination and to identify areas where groundwater is more susceptible to pollutants originating from human activities. Unlike methods that evaluate only the intrinsic vulnerability of aquifers, POSH focuses on the interaction between natural characteristics and anthropogenic pressures, making it particularly suitable for regions with intensive land use, such as agricultural or urban areas. This method is based on two key attributes: (a) the origin of the pollutant and (b) its hydraulic load. In this study, only point sources of contamination were considered and classified into three risk levels: low, moderate, and high.

Visual identification of activities associated with potential contamination sources was carried out using Geographic Information System (GIS) tools—specifically the open-access software Google Earth Pro and QGIS. Satellite imagery interpretation and industrial registry data from the Federation of Industries of the State of Paraíba (FIEP) were used to locate and characterize these sources. The investigation focused on identifying industries, gas stations, landfills, cemeteries, and auto repair shops. These sources were mapped in accordance with POSH method guidelines for activities that present a potential risk of contaminating groundwater.

Table 2 shows the classification of point sources of contamination adopted in this study, following the POSH method. An inventory of all identified activities was compiled into a database, based on the subsurface contaminant load potential and the contamination sources associated with anthropogenic activities. After identifying these potential sources, the risk of groundwater contamination was evaluated using the POSH framework, considering the specific type of activity present in the study area.

**Table 2:** Classification of point sources of contamination according to the POSH method

Potential	Point sources					Value
	Solid waste disposal	Industrial areas	Wastewater lagoons	Other (urban)	Mining and oil exploration	
Very high	Industrial waste of unknown origin	Type 3 industry or any activity handling > 100 kg/day of hazardous chemicals	All type 3 industrial waste; any effluent (except domestic sewage) if area > 5 ha	Gas stations, transport routes with regular hazardous cargo	Oil field operations, metal mining	10
High	Industrial waste of known origin	Type 2 industry	Domestic sewage if area > 5 ha	Gas stations, transport routes with regular hazardous cargo	Some inert material mining activities	8
Moderate	Rainfall > 500 mm/year with residential, agro-industrial, or industrial waste	Type 2 industry	Domestic sewage if area > 5 ha; other non-specified cases	Cemeteries	—	6
Low	Rainfall < 500 mm/year with residential, agro-industrial, or type 1 industrial waste	Type 1 industry	Mixed wastewater from residential, urban, agro-industrial, or non-metal mining	—	—	4
Very low	Rainfall < 500 mm/year with residential, agro-industrial, or type 1 industrial waste	Type 1 industry	—	—		2

### Land use and land cover changes in Campina Grande

Land use and land cover changes in Campina Grande were analyzed using land cover maps from the MapBiomas Project (2019) via the Google Earth Engine (GEE) platform. A detailed description of the project is available at http://mapbiomas.org. MapBiomas provides annual time series of land cover and land use derived from the classification of Landsat 5, 7, and 8 images, with a spatial resolution of 30 m. The classification process employs a semi-automated machine learning approach based on Random Forest algorithms to generate land cover and land use maps. The GEE procedures involved importing the MapBiomas layers corresponding to the selected years, filtering them by land use classes, and developing JavaScript scripts to extract area statistics per class, generate annual thematic maps, and calculate temporal change indices. Consequently, detailed annual maps have been produced since 1985. In this study, classified maps for Campina Grande were used for the years 1995, 2005, 2015, and 2022. All images were obtained in GeoTIFF format, using the UTM projection and WGS-84 datum. Seven land cover classes were considered: (a) Forest, (b) Caatinga, (c) Grasses, (d) Pasture, (e) Urban, (f) Exposed soil, and (g) Water.

#### Analysis of land use and land cover change dynamics

To assess the annual rate of change in land use types during the study period, the single land use change index (*K*) proposed by Xiao et al. (2006) and employed by Wu et al. (2016) was applied. The model is described by the following equation:

2 $$K=\frac{\left({A}_{t}-{A}_{a}\right)}{{A}_{a}T}100$$

where *A*_*a*_ represents the land-use area (in km^2^) at the earlier reference year and *A*_*t*_ represents the area at time *t* (most recent year), and *T* is the total number of years analyzed.

To assess urban expansion during the study period, the urban expansion index (*SI*) was used, as defined by Tian et al. (2005):

3 $$SI=\frac{\left(L{U}_{t}-L{U}_{t-1}\right)}{LT}100$$

where *SI* is the urban expansion index for a specific grid cell during the period, *LU*_*t*_ is the land use area at time *t* (current year), *LU*_*t*−1_ is the land use area at time *t* − 1 (previous year), and *LT* is the total area.

The SI is categorized into five development intensity levels, as follows: *SI* < 0.001%, unchanged area; 0.001% ≤ *SI* < 0.1%, insignificant development; 0.1% ≤ *SI* < 1%, rapid development; 1% ≤ *SI* < 5%, accelerated development; and *SI* ≥ 5%, extreme development.

### Groundwater contamination risk mapping

Based on the spatial distribution of well data, maps of the *G*, *O*, and *D* indices were generated, followed by the development of the natural groundwater vulnerability map for the study area using the inverse distance weighting (IDW) interpolation method. Subsequently, a groundwater contamination risk map was produced by integrating the results from the GOD method (representing the natural vulnerability of the aquifer) with those from the POSH method (representing anthropogenic pressures on the aquifer) (Table 3). This approach allowed for a comprehensive, integrated risk assessment.

**Table 3:** Cross-combination of GOD and POSH methods for identifying groundwater contamination risk zones

Risk level	GOD value	POSH value	Classification
Very high	10	10	Very critical
Alto	8	8	Critical
Moderate	6	6	Moderate
Low	4	4	Low
Very low	2	2	Insignificant

## Results and discussion

### Spatiotemporal analysis of wells in Campina Grande

**Fig. 5:**
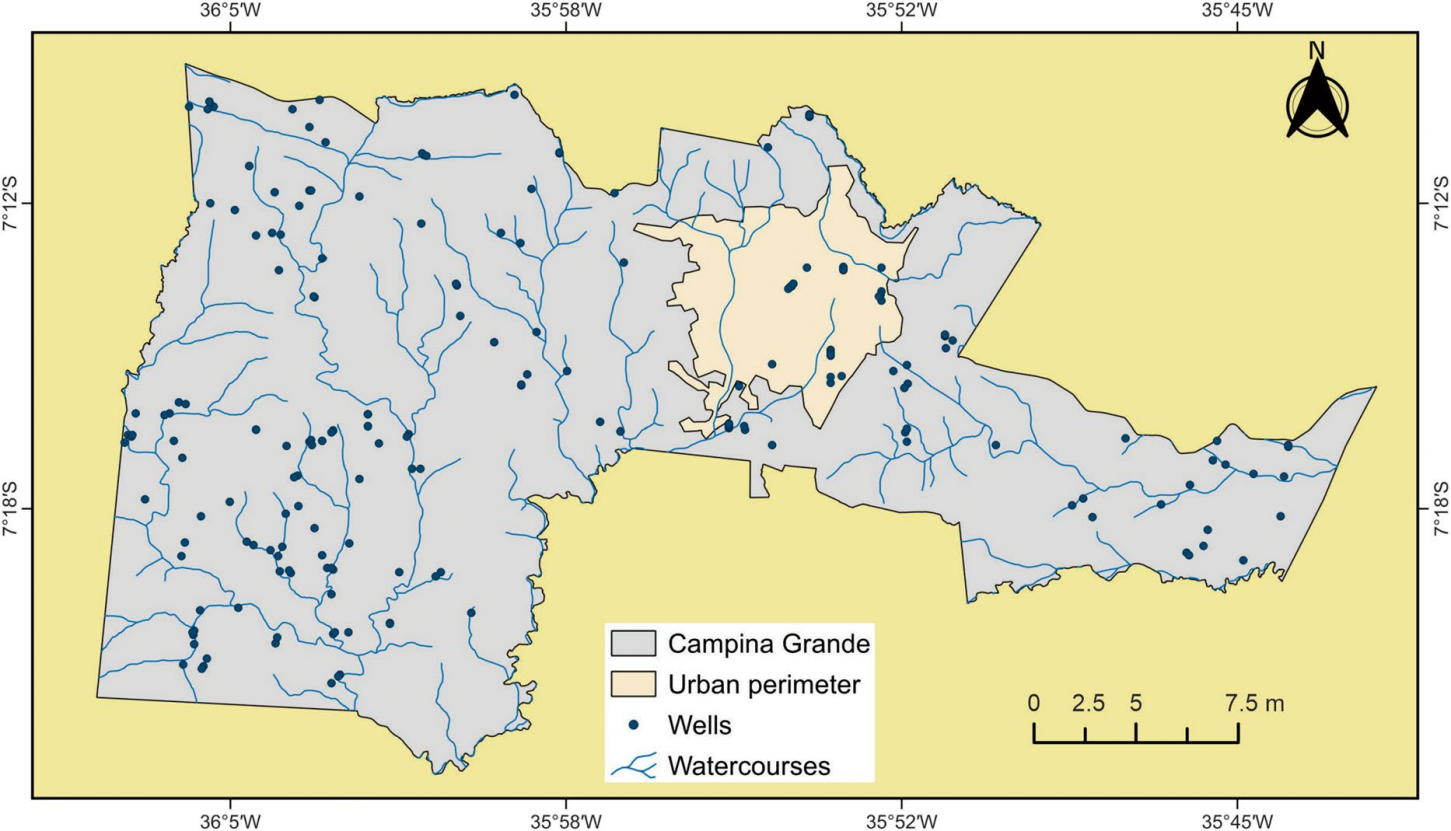
Wells exploited during the periods 1995–1999, 2000–2009, and 2010–2022

The results highlight the importance of integrating land use and well data with geospatial mapping tools for assessing groundwater vulnerability and contamination risk. In this context, the study identified areas more susceptible to contamination, considering both the natural characteristics of the subsurface water reservoirs—which influence the likelihood of pollutant infiltration—and anthropogenic factors related to land use in areas that pose a pollution risk to groundwater sources. Campina Grande has a well-documented history of groundwater use. To understand this dynamic, the spatial distribution of wells was analyzed to illustrate the temporal evolution of groundwater abstraction (Fig. 5).

Between 1995 and 2009, groundwater extraction occurred predominantly in rural areas of the municipality—such as farms, settlements, and smallholdings—with few exceptions in the urban perimeter. This pattern indicates the necessity and dependence of rural populations on groundwater for a range of purposes, including agriculture, livestock, and human consumption, contributing to improved water availability for these activities.

In contrast, the number of wells drilled in the urban area significantly increased from the 2000 s onward. These wells have supported various sectors, including industry, commerce, public services (municipal and state agencies), and general users. Notably, many of the wells were located near watercourses, suggesting a relationship between surface and groundwater systems, especially in terms of aquifer recharge processes. Furthermore, recognition of geotectonic features such as faults and fractures within the crystalline basement is essential, as groundwater occurrence in these structures is critical for successful abstraction. The spatiotemporal distribution of wells is summarized in Table 4, which presents the historical record of groundwater abstraction and the number of wells drilled in each time interval.

**Table 4:** Historical record of groundwater abstraction through wells in Campina Grande

Period	Number of wells drilled
1995–2000	105
2001–2004	56
2005–2008	9
2009–2012	12
2013–2016	55
2017–2020	71
2021–2022	21

The period between 1995 and 2000 witnessed a marked increase in well drilling, largely associated with severe drought events. Another significant phase of groundwater exploitation occurred from 2013 to 2020, which coincided with water supply crises (particularly from 2014 to 2017) and the lack of effective water resource management during dry years.

It is important to note that the identified wells do not include unregistered or clandestine wells, which pose a serious threat to public health due to the absence of monitoring and regulation. These unlicensed wells jeopardize the quality and functionality of legally registered wells, as they may cause contamination or other impacts despite the latter meeting regulatory standards.

### Vulnerability assessment

The vulnerability index was assessed based on the 199 groundwater abstraction points identified in the study area. Accurate identification of each variable and assignment of the corresponding value were essential to determine the natural vulnerability of the aquifer system.

#### G parameter

**Fig. 6:**
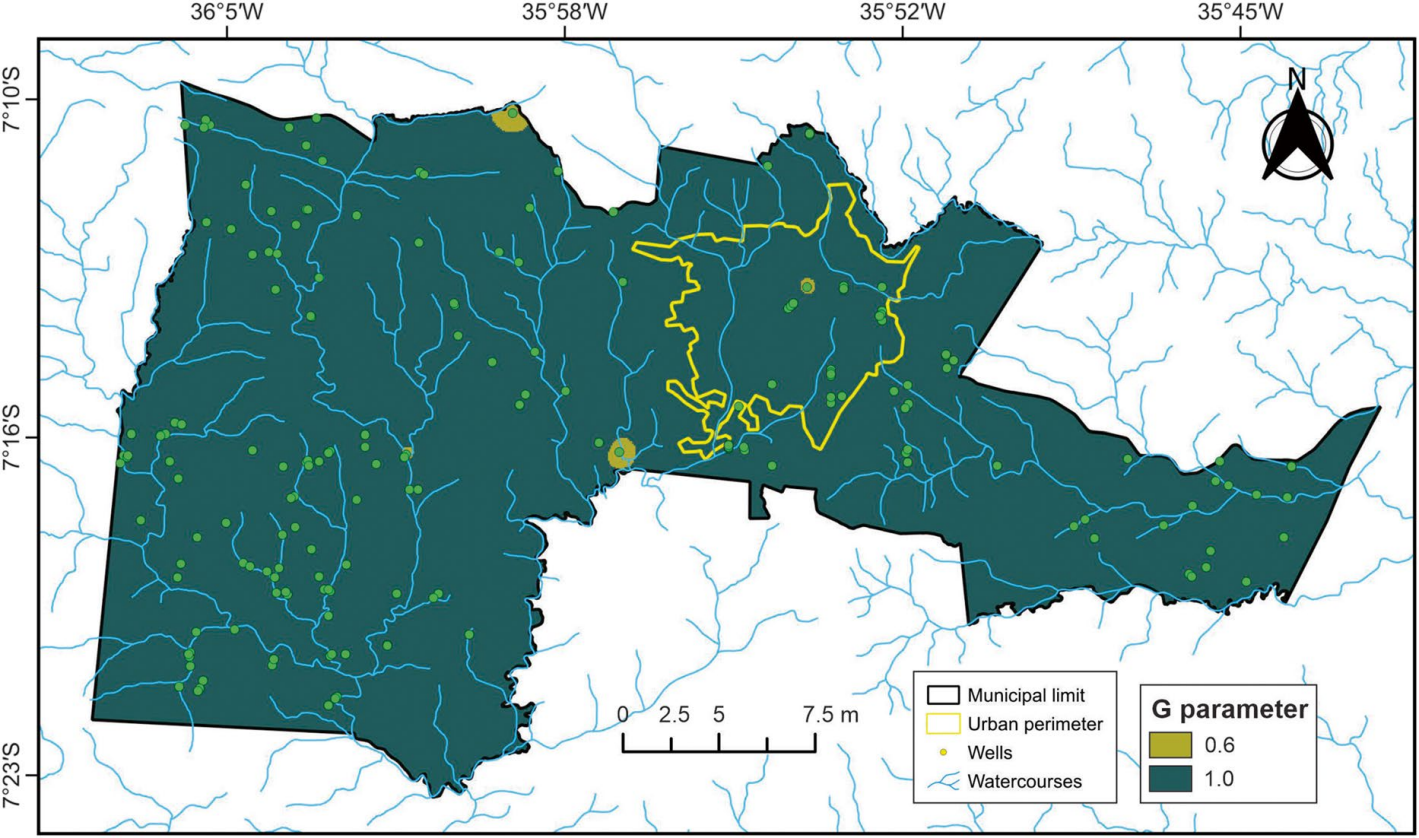
Map of the *G* parameter (degree of groundwater confinement)

Determining the degree of confinement (*G* parameter) is fundamental for evaluating aquifer vulnerability in a given region. This is because the ability of contaminants to infiltrate into the unsaturated zone depends on rainfall patterns and the associated transport of solutes and particulates (Lima et al., 2023). Based on this principle, the degree of hydraulic confinement in the study area was classified into two types: unconfined aquifer and covered unconfined aquifer. The unconfined aquifer, in which groundwater is under atmospheric pressure, comprises approximately 97% of the study area and was assigned a vulnerability index value of 1.0. This type of aquifer lacks impermeable layers above it, providing no separation between surface and groundwater. As shown in Table 5 and Fig. 6, this condition dominates the area and implies that contaminants can readily infiltrate the subsurface and reach the aquifer.

**Table 5:** Summary of well confinement types and their corresponding indices

Degree of confinement	Assigned index	Number of wells
Unconfined	1.0	193
Covered unconfined	0.6	6

The covered unconfined aquifer accounts for the remaining 3% of the study area and was assigned a value of 0.6, indicating a lower degree of vulnerability compared to fully unconfined aquifers. This type of aquifer is overlain by a protective layer—such as soil, sediment, or organic material—which, while not fully impermeable, offers a certain level of protection. The presence of this cover can delay the infiltration rate of contaminants and reduce the speed at which pollutants reach the water table.

The predominance of unconfined aquifers over covered unconfined systems suggests a significantly higher risk of rapid contamination across most of the study area. This information is critical for groundwater management and planning, particularly in the development of risk mitigation strategies aimed at protecting groundwater quality.

#### O parameter

**Fig. 7:**
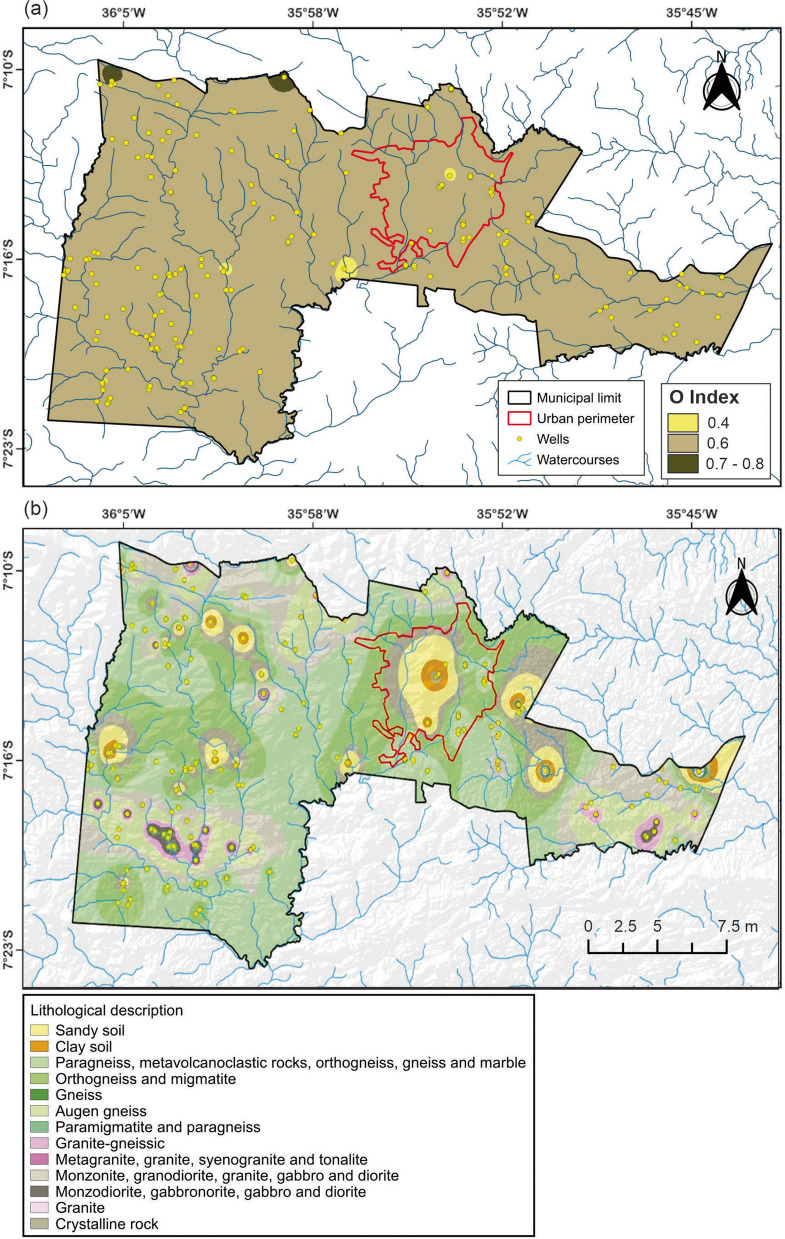
**a** Map of lithology and consolidation degree of the vadose zone (*O* parameter); **b** simplified lithological map of Campina Grande, based on data from well reports in the SIAGAS system and the geological map of the Borborema Province

Figure 7a presents the map of lithology and consolidation degree of the vadose zone or confining layers, corresponding to the *O* parameter. The soil and lithological materials located above the saturated zone of the aquifer influence both the contaminant travel time and the physical–chemical processes responsible for attenuation (Cutrim & Campos, 2010). These overlying strata either facilitate or inhibit the movement of potentially harmful fluids that may compromise subsurface water quality (Carvalho et al., 2020). The values observed for the *O* index in this study range from 0.4 to 0.8 (Table 6).

**Table 6:** Soil and lithology types associated with wells and their respective GOD index values

Soil/lithology	Assigned index	Number of wells
Gravel	0.8	1
Crystalline rocks	0.6	193
Sandy soil	0.6	1
Clayey soil	0.4	4

The results indicate a predominance of hard consolidated rocks throughout most of the municipality, with small areas of unconsolidated sediments (e.g., sand and clay), as shown in Fig. 7b. The soils found at the groundwater abstraction points selected for this study include clayey and sandy materials, distributed in small portions of the territory. The presence of sandy soils suggests a higher contamination potential due to their larger grain size and higher infiltration capacity when compared to clayey soils, which are generally more compact and less permeable. Additionally, traces of gravel were also identified in isolated locations. Crystalline rocks—both igneous and metamorphic—were found to predominate across the entire municipality.

The simplified lithological map was constructed based on the characteristics of geological profiles reported in technical documents from SIAGAS and the geological map of the Borborema Province, as compiled by Santos et al. (2021). The predominance of crystalline rocks may facilitate direct contaminant entry in areas with significant fractures and fissures, particularly where no thick overburden layers exist to act as a natural barrier. It is important to emphasize that not all subsurface layers and geological profiles offer the same degree of attenuation for contaminants. Aquifers tend to be more vulnerable to pollution in locations where consolidated rocks present high fracture density (Galvão et al., 2015).

#### D parameter

**Fig. 8:**
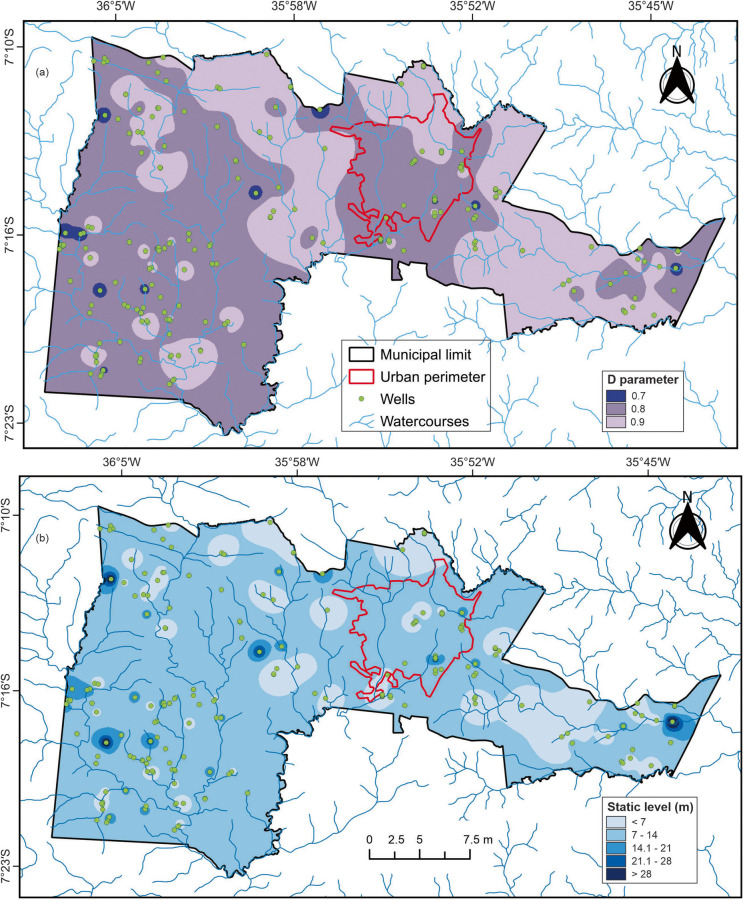
**a** Map of the distance to the water table (*D* parameter); **b** map of static water levels for the wells selected for the GOD method

The *D* parameter (Fig. 8a) represents the vertical distance from the ground surface to the groundwater level, defined by the static water level, which is measured in meters while the well is at rest. The *D* index values in this study ranged from 0.7 to 0.9. The areas classified as having higher natural vulnerability to contamination are distributed across most of the study area and are represented by index values of 0.8 (58% of the data), corresponding to depths between 5 and 20 m, and 0.9 (35% of the data), which corresponds to shallow depths (< 5 m) (Fig. 8b).

Static water level values varied from less than 7.6 m to greater than 28.2 m. However, most wells were concentrated around 4 m in depth. The deepest recorded water level was 46.2 m, observed at a single groundwater abstraction point located in the eastern portion of the study area, representing only 0.5% of the dataset. The mean static water level was 9.8 m, indicating a predominance of shallow aquifers (Table 7). According to Fig. 8b, the depth range between 7.6 and 14.5 m covers the largest portion of the study area. It is important to emphasize that shallower groundwater levels increase the likelihood and speed with which contaminants from the surface may reach the saturated zone, thus contributing to higher vulnerability to pollution.

**Table 7:** Descriptive statistics of the static water levels of the investigated wells

Statistic	Static water level (m)
Mean	9.8
Mode	4.0
Median	8.5
Maximum value	46.2
Minimum value	0.8

#### GOD index

**Fig. 9:**
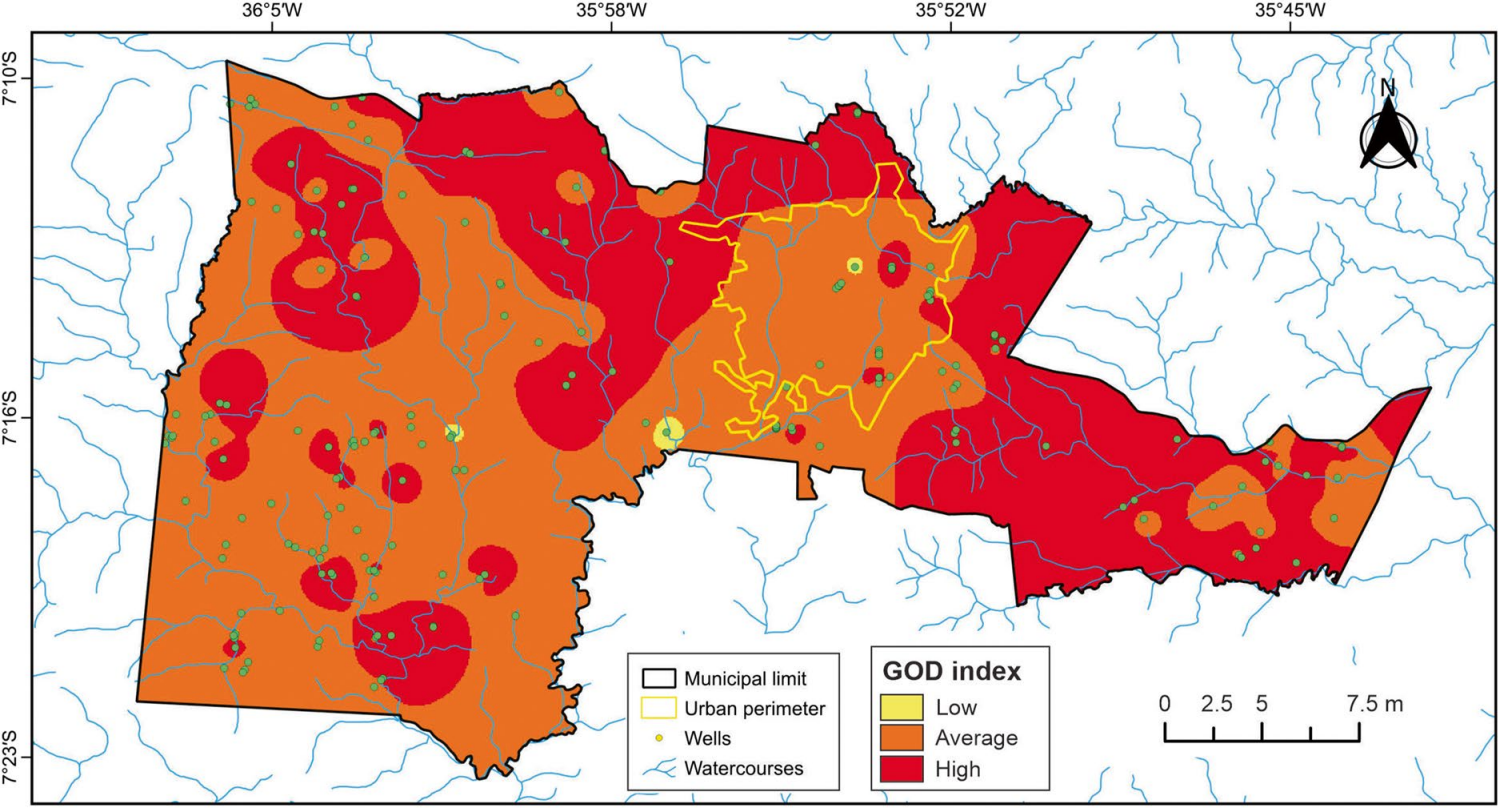
Natural vulnerability map of the aquifer to contamination based on the GOD index

The groundwater vulnerability map generated using the GOD index (Fig. 9), derived from the multiplication of the previously analyzed parameters (*G*, *O*, and *D*), identified three vulnerability classes: low, moderate, and high. Each classification reflects specific characteristics of the aquifer system under different contamination scenarios and should be interpreted accordingly.

Certain areas, primarily located in the eastern and northern portions of the study area, exhibit high vulnerability, accounting for 33% of the analyzed data. These zones often coincide with shallow water tables and the presence of fractures and fissures in the crystalline rock matrix, which likely facilitate rapid contaminant infiltration. Consequently, these areas are vulnerable to a wide range of contaminants under various pollution conditions.

The majority of the study area (65%) was classified as having moderate vulnerability, meaning the aquifer is susceptible to some contaminants—but primarily under conditions of sustained leaching or continuous pollutant input, such as those associated with solid waste accumulation. The urban perimeter of Campina Grande falls predominantly within this category, highlighting the combined effect of anthropogenic activities (as shown in Table 2) and the natural susceptibility of the aquifer to contamination.

Only 2% of the study area presented low natural vulnerability, indicating that, although these zones are less susceptible, they may still be affected by persistent conservative pollutants over the long term. This finding underscores the importance of continued monitoring and control, even in areas with apparently low vulnerability.

The assessment of natural vulnerability in groundwater reservoirs is of substantial relevance for identifying areas at risk of contamination, especially in regions with environmental and economic significance (Jain, 2023). In this context, the territorial management of Campina Grande must acknowledge that once groundwater becomes contaminated, it poses risks to human health, and the remediation process is typically complex, costly, and time-consuming.

### Recent analysis of land use and land cover in Campina Grande

Table 8 presents an analysis of the area occupied by various land use categories in Campina Grande over the studied years, along with values for the K metric (land use change rate) and S metric (urbanization rate). These metrics are essential for understanding landscape transformations and the evolution of urbanization in the region. By examining the occupied area for each land use category over time, it is possible to identify patterns and trends that reflect the dynamics of local development. Variations in land use types—such as urban, agricultural, forested, and others—offer insights into long-term changes.

**Table 8:** Land use types in the study area over the analyzed years, with corresponding K and S values

**Land use**	**Area (km**^2^)				**K (%)**
	**1995**	**2005**	**2015**	**2022**	
Forest	2.70	1.63	0.98	2.08	−1.622
Caatinga	160.75	152.69	165.39	149.90	−0.250
Grasses	0.76	0.66	1.79	0.37	−1.901
Pasture	241.17	265.37	253.53	250.81	−0.099
Urban	58.50	61.07	66.22	78.21	1.248
Exposed soil	2.82	1.42	2.05	3.49	0.880
Water	2.72	3.71	2.03	2.10	−0.844
**Land use**	**Urbanization index (S) (%)**				
	**1995–2005**		**2005–2015**		**2015–2022**
Urban	0.53		0.85		2.67

The K metric is used to quantify and interpret changes in land cover. Positive K values indicate an increase in urban or other specific land uses, while negative values suggest a reversion to more natural uses, such as forests or agricultural land. The S metric, representing the urban expansion index, directly measures the rate of urban growth. Increasing values of S reflect accelerating urbanization, whereas decreasing values may indicate a stabilization or even a decline in urban influence.

The combined analysis of K and S provides a comprehensive view of land use and urbanization changes throughout the studied periods. These data are valuable for guiding sustainable planning policies, environmental conservation strategies, and effective management of natural resources. The ability to monitor and interpret these changes over time supports a proactive approach to achieving a balance between human development and environmental preservation in the region.

The analysis of land use variations reveals distinct patterns of change over time. The Forest class experienced a significant decline, possibly due to deforestation or environmental degradation. The Caatinga ecosystem showed a slight reduction, indicating mounting pressure on this semiarid vegetation. Similarly, the Grasses category decreased, likely reflecting changes in natural vegetation cover.

The Pasture category also declined slightly (−0.099%), suggesting potential changes in agricultural practices or land use preferences. In contrast, Exposed soil showed a moderate increase (0.88%), likely driven by the expansion of the Urban category, which increased by 1.248%. These trends reflect dynamic land transformations and highlight the ongoing tension between human development and environmental conservation.

**Fig. 10:**
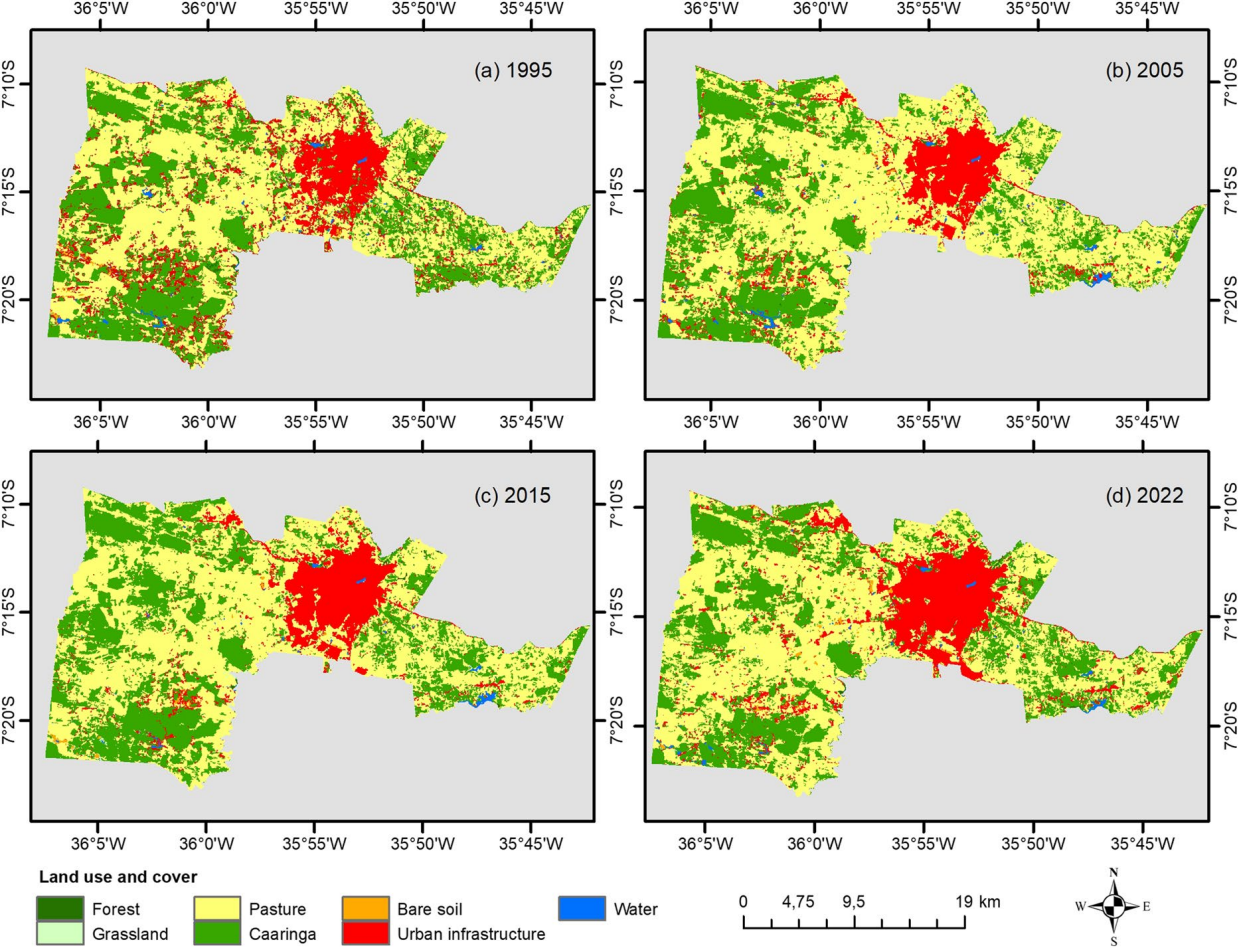
Land use maps for Campina Grande for the years: **a** 1995, **b** 2005, **c** 2015, and **d** 2022

Figure 10 presents a visual analysis of land use in Campina Grande for the years 1995, 2005, 2015, and 2022, offering a spatial perspective on urban and rural landscape changes. In 1995, Campina Grande appeared less urbanized, with more extensive areas of forest, Caatinga, and pasture—indicating a predominantly non-urban configuration. By 2005, changes in land distribution and urban growth became evident, possibly associated with increased infrastructure and residential expansion.

According to Silva et al. (2021), between 1980 and 1991, Campina Grande experienced a population increase of 78,480 inhabitants. However, in the subsequent decade (1991–2000), population growth slowed, with an increase of only 29,024 inhabitants—an overall growth rate of 8.16%. The period 2015–2022 recorded the highest urban expansion index (2.67%), surpassing previous intervals.

This deceleration in population growth is attributed in part to the increasing centrality of João Pessoa, the state capital, a process reinforced by national policies favoring state capitals since the 1970s. In addition, economic centralization in southeastern Brazil and the lack of new stimuli in the local economy contributed to the declining growth rate. The decline in rural population further emphasizes the continuous urbanization process, reflecting not only the urban pull but also structural economic transformations that directly influence demographic distribution. Thus, the interplay between economic, political, and demographic factors emerges as a key element in understanding population and urban dynamics in Campina Grande over recent decades.

The results for 2015 suggest continued urbanization, with expanded urban areas and observable shifts in land occupation patterns. Factors such as population growth, economic development, and urban planning policies likely contributed to these transformations. The 2022 map provides an updated view of land use distribution, highlighting ongoing urbanization trends and landscape alterations. This recent perspective supports informed urban planning and sustainable growth management in Campina Grande. In summary, Fig. 10 visually illustrates the evolution of land use in Campina Grande over the past decades, emphasizing the complex interactions between urban growth and rural land dynamics. It serves as a valuable tool for researchers, urban planners, and policymakers engaged in sustainable land management and regional development.

### Contamination risk assessment

#### Point sources

Estimating the risk of subsurface water contamination in Campina Grande provides essential support for territorial management that aligns with environmental and societal needs. The identified sources (Table 9) were classified according to the three risk levels established by the POSH method—low, moderate, and high—guiding technical criteria for decision-making concerning well drilling near potential pollution sources.

**Table 9:** Potentially polluting activities identified in the municipality of Campina Grande

Activity	Quantity	POSH index
Electronics industry	1	High
Chemical manufacturing	2	Very high
Plastics manufacturing	4	Very high
Metal processing industry	6	Very high
Auto repair shops	175	Elevado
Sanitary landfill	1	High
Soap and detergent industry	3	Moderate
Pulp and paper industry	4	Moderate
Textile industry	4	High
Cemeteries	10	Very low
Fuel stations	65	High
Car wash stations	74	High
Food and beverage industry	5	Low

**Fig. 11:**
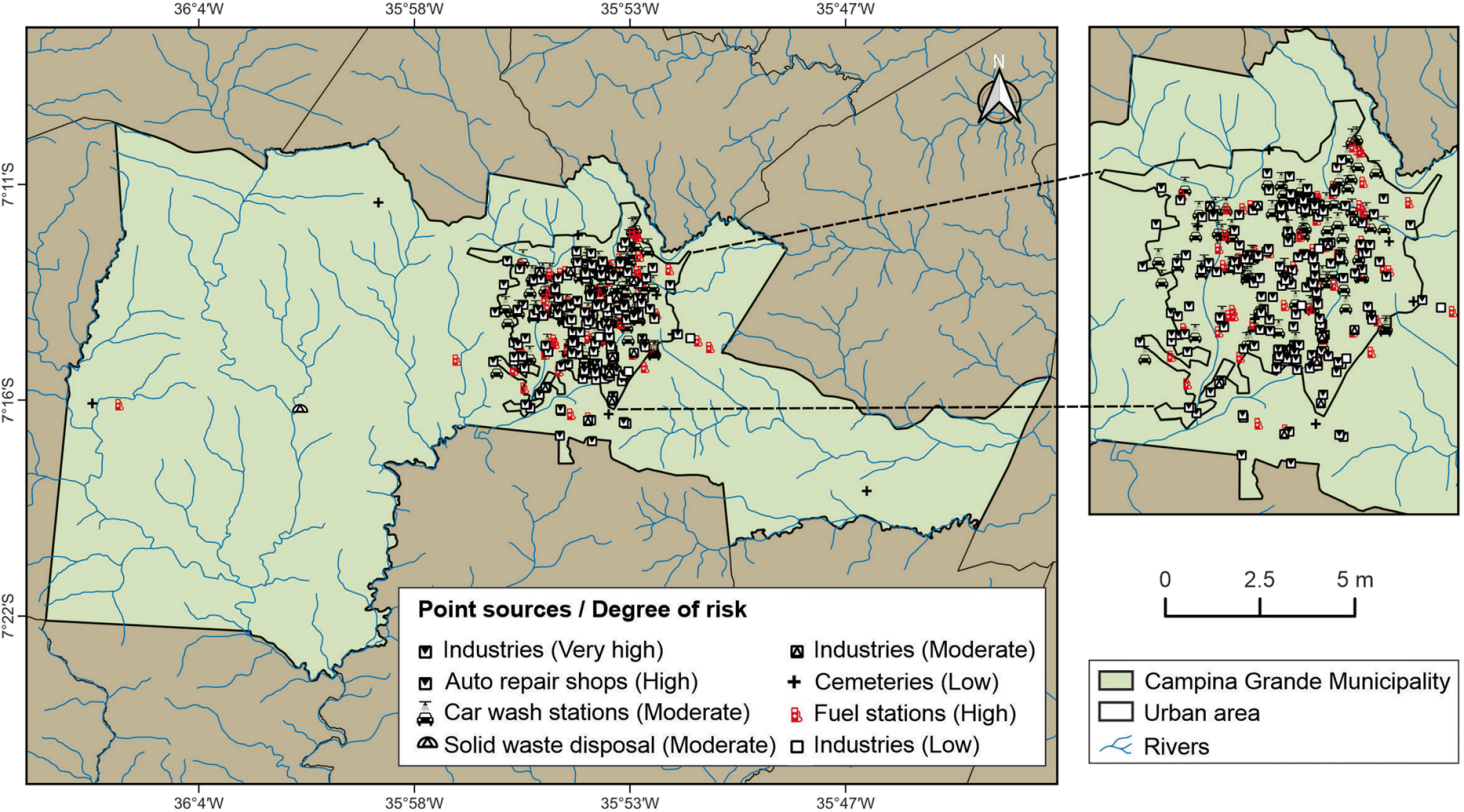
Map of point sources of contamination in the municipality of Campina Grande

Campina Grande is one of the main industrial hubs in the state of Paraíba. This context demands close attention to the disposal of pollutants generated by industries that handle aggressive chemical substances. These compounds, when in contact with groundwater, can pose significant contamination risks—especially in cases involving high-risk pollution sources lacking appropriate waste treatment. The spatial distribution of contamination-prone activities (Fig. 11) is concentrated predominantly in the urban area and its immediate surroundings, with few isolated sources located in rural zones.

A high concentration of industries with elevated contamination potential is found in the southern portion of the urban area, particularly in the industrial district (highlighted in Fig. 11). These include chemical, plastics, electronics, and metal processing industries, which together account for 3.6% of all identified sources. Even small quantities of toxic and persistent chemicals can generate contamination plumes in groundwater, especially in aquifers characterized by high flow velocities. Substances commonly associated with these sectors include halogenated hydrocarbons, aromatic and chlorinated compounds, phenols, alkylbenzenes, and heavy metals (Lapworth et al., 2012).

These chemical compounds can pose serious public health risks, particularly when not properly handled by the industries that use or generate them. Effluent disposal is another critical issue—inadequate control or treatment significantly increases the likelihood of contamination. In Campina Grande, the industrial district is located at some distance from residential neighborhoods, which could suggest a degree of protection for groundwater sources supplying those communities. However, the continued expansion of the urban area raises concerns, as well as exploitation in newly urbanized zones may encounter residual contamination from nearby industrial activities.

Auto repair shops represent the most numerous categories of identified sources, accounting for 49.4% of the dataset. These facilities are distributed throughout the city and are classified as high-risk pollution sources. In the southernmost part of the urban perimeter, a cluster known as the mechanics’ district hosts a substantial concentration of such establishments. These workshops perform various services involving hazardous chemicals, which, when infiltrated into the subsurface, can interact with groundwater and pose serious risks to human health.

According to Rahmawati et al. (2024), an analysis conducted on several wells in the mechanics’ district revealed the presence of hydrocarbons, oils, and greases in the water table. The contamination observed was attributed to the mobilization of pollutants during the rainy season, which infiltrate through fractures in the soil and rock, ultimately reaching the aquifer. This suggests that areas with a high density of auto repair shops are more vulnerable to groundwater contamination than areas with isolated facilities. In addition to these high-risk sources, industries identified as having moderate contamination potential—such as textile factories, pulp and paper mills, and soap and detergent manufacturers—account for 3.2% of the total. The effluents associated with these industries include dyes (textile sector); various chemical, physical, biological, and physicochemical pollutants (pulp and paper); and persistent chemical compounds from detergent production. These substances, if improperly managed, can also pose significant risks to public health (Fazzo et al., 2017).

Another area of concern is the municipal sanitary landfill, classified as a moderate contamination source (accounting for 0.2% of the identified data), located in the western part of the municipality. In this area, there is a significant possibility that leachate—the liquid produced by the decomposition of waste—may infiltrate the subsurface and increase the risk of groundwater contamination. Although the landfill is located at some distance from the urban zone, it is important to note that wells situated near this type of facility remain at risk of severe contamination.

According to Freeze and Cherry (1979), when sanitary landfills are located on substrates with moderate permeability—such as sand, gravel, or fractured rock—the migration of leachate can lead to contamination over areas far beyond the landfill boundaries.

Fuel stations located in the municipality under investigation are classified as moderate-risk contamination sources. This study identified 65 fueling stations, representing 18.3% of the total point sources analyzed. A significant number of these establishments operate in the urban area, indicating widespread potential for groundwater contamination.

Oil and gasoline have lower densities than water and are immiscible, meaning they do not mix with water (Freeze & Cherry, 1979). As a result, when spills or leaks occur, these substances tend to migrate directly into the unsaturated zone, where they may remain for extended periods or eventually reach the water table.

It is likely that storage tanks older than 20 years may exhibit structural corrosion and be prone to significant leaks—unless they receive regular maintenance. Furthermore, pipelines connecting tanks to fuel pumps are susceptible to rupture due to heavy vehicle traffic or poor installation quality. For this reason, it is essential to conduct regular integrity tests on tanks and pipelines to detect potential leaks and prevent further groundwater contamination (Żychowski & Bryndal, 2015).

Cemeteries located in Campina Grande (representing 2.9% of the identified point sources) are distributed across both urban and rural areas, differing in terms of ownership (public and private). According to Żychowski and Bryndal (2015), cemeteries typically generate a relatively small microbiological pollution load, generally confined to a limited area. This impact can be mitigated through the use of impermeable tombs or corrosion-resistant coffins.

However, this study found that a significant number of cemeteries are situated near watercourses, increasing the risk of pollution. Moreover, the presence of shallow water tables elevates the vulnerability of wells to contamination, particularly from necrochorume. As described by Fernandes et al. (2025), necrochorume is a viscous liquid denser than water, containing various pathogenic bacteria responsible for diseases such as tetanus, gas gangrene, foodborne infections, typhoid and paratyphoid fevers, bacillary dysentery, and hepatitis A.

According to the applied methodology, cemeteries are generally classified as low-risk point sources of contamination. However, given the aforementioned conditions, the risk of groundwater pollution increases. Therefore, in this study, cemeteries were reclassified as moderate-risk point sources. In this context, control measures are recommended to monitor potential clandestine groundwater abstraction near cemetery areas.

Car wash facilities were also included in this study due to their handling and disposal of environmentally harmful effluents, which may affect groundwater reservoirs. A substantial number of these establishments—mainly micro- and small-scale businesses—were mapped exclusively in urban areas, comprising 21% of the total identified sources.

In addition to excessive water consumption, vehicle washing generates significant volumes of waste, including surfactants (chemical agents that enhance the solubility of organic compounds in water or aqueous phases), soot, dust residues, gasoline, grease, and a wide range of automotive contaminants (Candeias et al., 2020).

According to CONAMA Resolution No. 362/2005, which regulates the collection and final disposal of used or contaminated lubricating oil, Article 12 explicitly prohibits the disposal of such oils into soils, subsoils, and water bodies, as well as sewage systems or wastewater drainage networks.

In this context, it is important to highlight that in 2015, the Water Management Executive Agency of Paraíba (AESA) identified 36 car wash facilities in Campina Grande operating unauthorized artesian wells, without the required license or water use permit. This discovery led to a series of official notifications during 4 days of inspections. AESA stated that noncompliance with the legal requirements for groundwater exploitation may result in fines, and in some cases, the sealing of hydraulic pumps used for water extraction (Governo da Paraíba, 2015, online).

Given these findings, car wash facilities were classified in this study as moderate-risk point sources of groundwater contamination. Therefore, compliance with current regulations is essential, particularly due to the risk of direct interaction between generated residues and the water table.

Finally, industries in the food and beverage sector, although comprising only 1.4% of the total identified activities, are considered low-risk point sources due to their relatively minor contaminant loads. Nevertheless, the persistence of even low-level pollutants may lead to gradual degradation of nearby groundwater reserves, particularly in the absence of proper control measures.

In total, 354-point sources of potential contamination were identified in the study area, distributed across low-, moderate-, and high-risk categories. This wide spatial distribution highlights the need for localized monitoring and the formulation of targeted mitigation strategies to ensure the protection of groundwater quality in Campina Grande.

## Advantages and limitations

The assessment of groundwater vulnerability and contamination risk is critically important for guiding preventive measures to be adopted by both governmental agencies and the private sector. Groundwater use must be closely monitored, especially considering the potential impacts of indiscriminate extraction on recharge zones. The predominance of moderate and high vulnerability classes in the study area—according to the GOD method—clearly delineates zones more prone to contamination, underscoring the need to regulate well development in these areas. Land use and land cover dynamics are essential considerations in this context, as they are directly linked to the emergence of contamination sources.

It should be emphasized that this study does not replace the need for complementary field investigations. Field validation could further improve the level of detail and accuracy. Nevertheless, all data used and analyzed in this research were obtained from reliable sources and based on established scientific methodologies, ensuring credibility and relevance. In recent years, methodological advances have enhanced both the GOD and POSH methods in mapping aquifer vulnerability and contamination risk. Key updates include optimized weighting schemes to reduce subjectivity, cross-validation with observational data, and hybrid approaches that combine classical indices with machine learning techniques to refine spatial predictions (Mehta et al., 2024; Ourarhi et al., 2024d). These improvements increase the robustness and predictive capacity of GOD and related models, particularly in regions with comprehensive geological and hydrogeological datasets.

For the POSH method, recent applications have emphasized the explicit quantification of pollutant loads and the incorporation of dynamic land use data, improving its sensitivity to temporal anthropogenic pressures—a critical advantage in areas undergoing rapid agricultural or urban transformation. Studies have demonstrated that when POSH is combined with land use time series and pollutant source inventories, it provides a risk assessment more directly linked to actual contamination sources (Campoverde-Muñoz et al., 2023).

Despite these advances, limitations remain. These include dependence on high-quality input data, uncertainties associated with spatial interpolation, and the risk of overfitting when machine learning models are applied without independent validation. Recent studies recommend the use of multi-method approaches (e.g., GOD + POSH + statistical validation) to offset individual biases and generate more reliable maps (Yuan et al., 2024).

Compared with these evolving approaches, the framework adopted in this study—integrating GOD and POSH—remains relevant because it captures both intrinsic vulnerability (GOD) and anthropogenic pressures (POSH). For greater applicability, future work should incorporate optimized weighting schemes, ROC/AUC validation, and land use time series (e.g., MapBiomas/GEE), as well as quantify spatial uncertainties to guide transparent management decisions (Fannakh & Farsang, 2022).

The GOD method, however, presents certain limitations. In regions with moderate variations in vulnerability, it often produces relatively homogeneous distributions, potentially masking local differences. Consequently, this method is more suitable for areas exhibiting strong contrasts in vulnerability levels (Fannakh et al., 2025). Another limitation is its restricted ability to represent the natural heterogeneity of subsurface systems; factors such as the complexity of the unsaturated zone and the presence of vertical wells pose additional challenges, particularly in karst environments (Taghavi et al., 2022).

The POSH method has proven valuable for assessing groundwater contamination risk (Díaz-Espíritu et al., 2024). Nonetheless, several limitations must be acknowledged. A key constraint lies in its reliance on accurate quantification of pollutant loads, which are often unavailable or only indirectly estimated, potentially reducing output reliability (Esquivel-Martínez et al., 2023). Moreover, its performance depends heavily on the quality and spatial resolution of land use and land cover data, limiting effectiveness in areas where such information is outdated or inconsistently monitored. Most critically, POSH does not incorporate the intrinsic vulnerability of aquifers, which may lead to overestimation of risks in areas subject to strong anthropogenic pressures but characterized by low natural susceptibility.

## Conclusions

This study assessed groundwater contamination risks and land use changes in the city of Campina Grande, located in the Brazilian semiarid region. The findings revealed that a lack of public awareness regarding aquifer overexploitation—combined with insufficient action by water management authorities to inform and engage users—hinders effective resource governance. Such negligence may compromise groundwater reserves, as uncontrolled extraction increases the likelihood of both contamination and depletion. The historical pattern of well drilling in the municipality highlights the growing demand for alternative water sources during periods of surface water scarcity. Notably, during the 2014–2017 water crisis, the number of wells registered in public databases increased significantly, reinforcing the need to recognize groundwater as a critical resource for ensuring water security and meeting societal needs.

The results, supported by land use data over the study period, show significant spatial changes, particularly in the urban fabric, with notable expansion from 2015 to 2022. This trend raises concerns regarding the preservation of remaining unoccupied areas. The study generated several positive outcomes: (i) data acquisition from publicly accessible systems; (ii) use of GIS environments to produce critical cartographic products; (iii) interdisciplinary integration of geography with geology and hydrogeology; (iv) demonstration of the need for efficient groundwater management; and (v) application of vulnerability and contamination risk assessment methods recognized by the scientific community.

Nonetheless, some limitations were identified: (i) the diversity of vulnerability and risk classifications complicates contaminant control, especially in urban areas; (ii) the presence of irregular or unlicensed wells limits monitoring and may place them near high-risk sources; (iii) incomplete data in many well drilling reports; (iv) lack of funding for fieldwork, which requires significant investment; and (v) the short duration available for this study, which constrained more in-depth analysis.

This research advances the scientific understanding of groundwater vulnerability by integrating intrinsic and anthropogenic factors through the combined application of the GOD and POSH methods. The proposed framework provides an effective approach for evaluating contamination risks in data-scarce semiarid regions, where aquifers serve as essential water sources for both urban and agricultural uses. By coupling hydrogeological indicators with land use dynamics derived from Google Earth Engine and MapBiomas data, this study demonstrates how geospatial analysis can enhance water resource management under conditions of climate variability and rapid urbanization. The findings have global relevance for semiarid regions facing similar environmental pressures, where population growth, industrial expansion, and recurrent droughts threaten groundwater sustainability. The methodological integration proposed here strengthens preventive management capacity, supports the United Nations Sustainable Development Goal 6 (Clean Water and Sanitation), and offers a transferable model for groundwater protection worldwide.

In summary, the findings of this study provide a comprehensive overview of the natural and anthropogenic factors that may affect groundwater quality in Campina Grande, identifying areas most susceptible to potential contamination. The integration of groundwater vulnerability assessment with land use dynamics enhances understanding of pollution sources and supports more robust decision-making. Therefore, this research can serve as a reference for future studies in the region. It is recommended that updates to well databases and the inventory of contamination sources be conducted periodically to reflect ongoing changes in the area.

## Data Availability

Data will be available on request.
